# Quality assessment of *Urochloa* (syn. *Brachiaria*) seeds produced in Cameroon

**DOI:** 10.1038/s41598-021-94246-w

**Published:** 2021-07-23

**Authors:** Njehoya Clémence-Aggy, Ntchapda Fidèle, Kana Jean Raphael, Etchu Kingsley Agbor, Sita R. Ghimire

**Affiliations:** 1 Institute of Research for Agricultural Development, P.O Box 2123, Yaounde, Cameroon; 2 Biosciences Eastern and Central Africa, International Livestock Research Institute, P.O. Box 30709-00100, Nairobi, Kenya; 3 Faculty of Science, University of Ngaoundéré, P.O. Box: 454, Ngaoundéré, Cameroon; 4 Faculty of Agronomy and Agricultural Sciences, University of Dschang, P. O. Box 222, Dschang, Cameroon

**Keywords:** Ecology, Plant sciences

## Abstract

*Urochloa* (syn. *Brachiaria*) is the most popular fodder of livestock farmers in Cameroon for hay and seed productions. Farmers in Cameroon have been producing *Brachiaria* seeds for decades for own uses and surplus are sold to neighbours, and to traders from Cameroon and neighbouring countries. However, there is no information available about qualities of these seeds. Fifteen *Urochloa* seeds samples were collected from farmers and/or government stations in five regions (Adamaoua, East, North, North West, and West) and analysed for major seed quality parameters along with seeds of improved *Urochloa* cultivar Basilisk imported from Brazil as a check. Study showed significant differences among treatments for various seed quality parameters tested (*P* < 0.0001). The highest thousand grains weight was recorded in Basilisk (5.685 g), followed by W12 (3.555 g), A05 (3.153 g) and N01 (2.655 g). Caryopsis number and caryopsis weight were highest in Basilisk followed by E09, A06, and W12. Of three conditions tested for seed germination, mean germination was the highest in greenhouse (7.39%) where Basilisk had the highest germination (25.5%) followed by N01 (18.50%), A05 (14.50%) and W12 (12.75%). The seed viability ranged from 18% (E09) to 81% (N01), and there were a positive and highly significant relationships between seed germination and viability traits (r = 0.883; *P* < 0.0001). This study showed a marked difference in seed quality parameters of *Urochloa* grass seeds produced in Cameroon, and the potential of developing *Urochloa* grass seed business in the Northern, Adamaoua and Western regions of Cameroon.

## Introduction

*Urochloa* grass is one of the most important tropical forages native to Africa. The genus *Urochloa* consists of about 100 documented species^1^ of which seven perennial species of African origin (*Urochloa arrecta, Urochloa brizantha*, *Urochloa decumbens*, *Urochloa dictyoneura, Urochloa humidicola*, *Urochloa mutica* and *Urochloa ruziziensis*) are used as fodder plants^2^. *Urochloa* grass was introduced to other parts of the world in different occasions^3–8^*. Urochloa* grass is the most extensively cultivated forage in tropical Americas^9–12^, and it is among the most widely cultivated forages in Asia, the South Pacific and Australia^13^.

In Africa, the evaluations of *Urochloa* grass for pasture improvement started in 1950s. Early studies confirmed a high biomass production and broad adaptation of some *Urochloa* species^14^. However, none of these *Urochloa* species were used for commercial pasture production in Africa essentially due to no role of sown pasture in the livestock production except in the smallholder dairies in the highlands^14^. A gradual intensification in the livestock production system in Africa has spurred demand for high quality feeds and improved forage including *Urochloa* grass. Improved forages have been one of the most preferred feed resources to livestock farmers as they provide quality feed option at a low cost and are resilient to the climate change.

The initial introduction and evaluations of improved *Urochloa* grass cultivars in Africa started in the early 2000s for ruminant nutrition, pest management (*push–pull-system*) and conservation agriculture^15^. Subsequently, additional hybrids and improved cultivars were introduced and evaluated for ruminant nutrition^16–18^. The Climate-smart *Brachiaria* Program (2012–2016) evaluated several improved *Urochloa* cultivars for adaptation, agronomy, and livestock performance in the East Africa. *Urochloa* grass when fed to livestock increased milk production in between 15–40% and there was over 50% increase in body weight in heifers^19,20^. Due to an excellent adaptation, desirable agronomic attributes, and substantial benefits to livestock productivity there is high demand for *Urochloa* grass from farmers, therefore many livestock development initiatives in the region have been promoting improved *Urochloa* grass. However, the pace of dissemination and adoption of *Urochloa* grass has been very slow in Africa due to unavailability and restricted access to seeds. Therefore, African farmers have been relying on rooted tillers as planting material from neighbouring farmers. This enables them to establish small-scale *Urochloa* pasture but the large-scale cultivation in wider geographical areas using rooted tillers is less practical as it requires substantial investment in buying, movement, and handling of bulky planting materials. Availability of rooted tillers in large quantity has been another bottleneck for wider adoption of *Urochloa* grass.

At present, *Urochloa* grass productions in Africa rely on imported seeds, and rooted tillers. A wide scale adoption of improved *Urochloa* grass is necessary to realize full benefits of this forage on livestock productivity in Africa. To achieve this, African farmers should have timely access to *Urochloa* seeds at affordable price. Regulatory restrictions on seed import, long-distance shipment, and small trade volume are major causes for high seed price and limited access to *Urochloa* seeds to farmers. A sustainable supply of *Urochloa* seeds in Africa requires lessening dependence on imported seeds and establishing an effective and commercial *Urochloa* seed production system in the continent. This proposition is realisable as other studies report success in *Urochloa* seed production in Cameroon and Kenya^21,22^.

In Cameroon, the introduction, and evaluations of *Urochloa* grass for fodder production started in the1960’s^23^. *Urochloa* is a popular forage promoted in Cameroon for hay and seeds production^22^. Farmers in Cameroon have been producing *Urochloa* seed for years for own uses and surplus seeds are sold to neighbours and vendors from other parts of the country and neighbouring countries like Nigeria and Central African Republic. *Urochloa* growers in Cameroon uses seeds rates of 50 to100 kg/ha, which is several folds higher than recommended seed rates of 3–7 kg/ha^24^. This practice of high seed rates used in Cameroon is likely due to a low-quality of locally produced *Urochloa* seeds. However, there is no information available on the qualities of *Urochloa* seeds produced in Cameroon. Therefore, this study aims to assess the qualities of *Urochloa* seeds produced in Cameroon and identify the suitable niches for *Urochloa* seed production in the country.

## Methods

### Description of the study area

**Figure 1:**
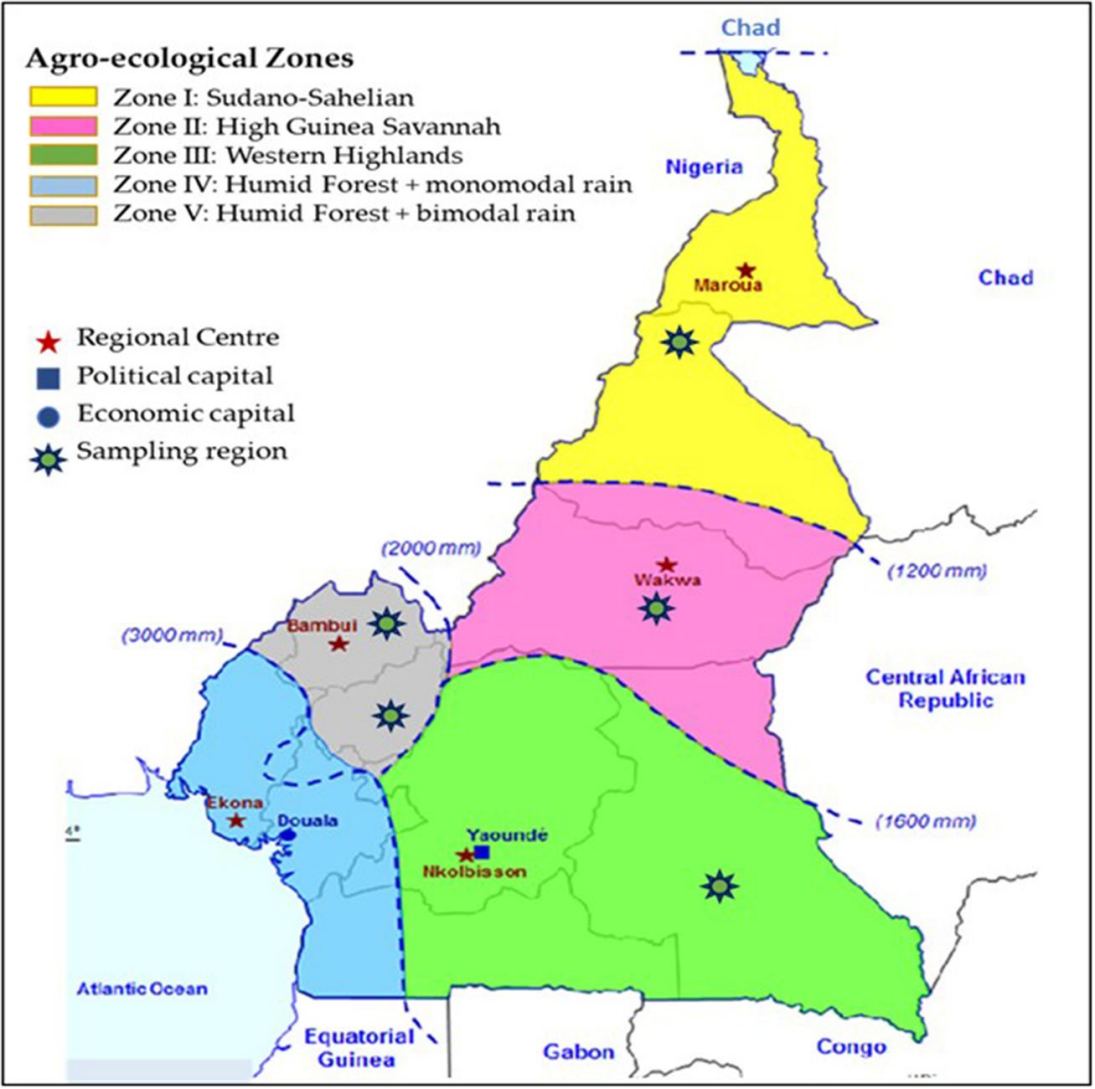
A map of Cameroon showing agroecological zones, regional centres, political and economic capitals and *Urochloa* seeds sample collection regions (https://agritrop.cirad.fr/556139/1/document_556139.pdf ).

Cameroon is located in between Equatorial Africa in the South and Tropical Africa in the North. It is also known as Miniature Africa due to a high geographical, climatic, and ecological diversities. It covers 475,650 km^2^ land mass and borders with Nigeria, Chad, Central African Republic, Congo Brazzaville, Gabon, and Equatorial Guinea. Cameroon can be divided into five agroecological zones; Sudano-Sahelian in the North and extreme North region, Sudano-guinea in the Adamaoua Plateau, Western High Plateau in West and North-west region, Humid Forest with unimodal rainfalls in the Littoral and South-west region, and the Humid Forest with bimodal rainfalls in Central and Eastern part of the country^25^ (Fig. 1). Livestock are important in the Western Highlands, Adamaoua Plateau and North^26^.

### Source of Urochloa seeds

*Urochloa* seed samples were collected from the North, Adamawa, East, West, and North-west regions that represent four agroecological zones in Cameroon. Selection of these regions was based on the history of *Urochloa* cultivation for hay and seeds production and the importance of the livestock. A total of 15 *Urochloa* seeds samples were collected from farmers and government’s agricultural stations and used for different analysis following the guidelines of Institute of Agricultural Research for Development (IRAD), Cameroon. The amounts of seeds collected per sample ranged in between 150 and 300 g. Although no official information available about *Urochloa* grass species/cultivar grown by farmers in Cameroon, it is most likely to be *U. ruziziensis* that has been vulgarized in the country following the early introduction and evaluations^22^. A recent agro-morphological characterization of four landraces of *Urochloa* grass from two regions of Cameroon has tentatively identified them as *U. ruziziensis* (Ntane Ojong et al. unpublished results) Other details about sample collection locality are presented in Table 1. *Urochloa decumbens* cv. Basilisk seeds imported from Marangatu Seeds, Brazil was used as a check in the study.

**Table 1:** Origin of *Urochloa* seeds samples collected in Cameroon for seed quality analysis.

Sample ID	Region	Sites	Altitude (m asl)	Rainfall (mm)	GPS Coordinates
N1	North	Garoua	248	1500	N09.35100/E013.28667
N2	North	Louguere	475	1500	N09.93060/E013.77282
N3	North	Louguere	388	1500	N09.93060/E013.77282
N4	North	Louguere	412	1500	N09.93060/E013.77282
A5	Adamaoua	Wakwa	1143	2000	N07.13600/E13.34600
A6	Adamaoua	Banyo	1250	2000	N06.28000/E11.33000
A7	Adamaoua	Banyo	1200	2000	N06.49000/E12.00000
A8	Adamaoua	Banyo	1250	2000	N06.74960/E11.80360
E9	East	Bertoua	717	2500	N04.34382/E13.41452
E10	East	Bertoua	717	2500	N04.35590/E13.43599
E11	East	Bertoua	717	2500	N04.343820/ E13.41045
W12	West	Bangangté	1205	3000	N05.10389/E10.33961
NW13	North West	Bambui	1288	3000	N06.01941/E10.05720
NW14	North West	Bambui	1601	3000	N05. 59767/E10.16859
NW15	North West	Bambui	1281	3000	N06. 01962/E10.05752

### Harvesting and processing of seed samples

All 15 seed samples from Cameroon were harvested manually in between November and December 2017. For seed samples W12, NW13, NW14 and NW15, inflorescences were cut at maturity, stored inside bag for three days and hand-shaken, whereas other eleven seed samples were collected by shaking inflorescence within a basket or bag. Seeds were manually cleaned and stored in jute bags on the bamboo ceilings or floors of houses at ambient temperature. In August, seeds were transported to the Bioscience eastern and central Africa—International Livestock Research Institute (BecA-ILRI) Hub, further cleaned to remove debris (if any), placed inside airtight plastic containers (Lock & Lock Interlock Door Pocket of one litre capacity). About 200 g seeds were put in each container and stored at 22 to 24 °C and ambient humidity of min. 58% and max. 75% until further analysis starting in September 2018. Otherwise stated, all empty to fully filled seeds in a sample were used in seed quality analysis. Basilisk seeds used as check in this study were produced in the State of Mato Grosso do Sul, Brazil in 2017 crop season and harvested at about 10% moisture content using tropical forages harvesting machine. Seeds were checked for qualities, processed using air and screens and gravity separator and stored in the warehouse. These Basilisk seeds were not scarified*.*

### Thousand grains weight

Manually cleaned seeds of each sample were counted into four batches of one thousand seeds and weighed on a digital balance (KERN PCB, Germany) for thousand grains weight (TGW) determination.

### Caryopsis percentage and weight

The caryopsis count was performed on 25 seeds per sample in four replicates. The seeds were counted in a batch of 25 seeds, placed in between wet double layer paper towels then kept inside a Ziploc plastic bag. The Ziploc bag was sealed and incubated in dark at room temperature for 44 h to soften the husk cover. Following the incubation each seed was dehusked by separating lemma and palea using forceps under dissecting microscope, and the presence/absence of endosperm/caryopsis was recorded. After the caryopsis count, caryopses were wrapped into a paper towel, dried at 40 °C for 72 h and weighed in precision digital balance (KERN PCB, Germany) for caryopsis weight determination.

### Seed germination test

Germination tests were performed in three different environments i.e., laboratory, greenhouse, and field. In laboratory tests, 100 *Urochloa* seeds of each seed sample was randomly arranged on a double layer paper towel in a petri dish in four replications. The paper towels were soaked with sterile water at saturation. Petri-dishes were placed into plastic bags, sealed, and incubated in dark at 28 °C. Germination data were recorded at 3, 7, 14 and 21 days after incubation. The cumulative germination data was used in analysis.

In greenhouse tests, a total of sixteen flat trays (48 cm × 16 cm × 15 cm) were filled with a mixture of sieved two parts of field soil and one part of well decomposed farmyard manure (volume/volume). One hundred seeds of each treatment were shown in a row at 2 cm depth, covered with soil and watered at saturation. Experiment was organized in randomized block design in four replicates. Trays were watered from the bottom as necessary, and germination data was recorded one week after seeding and at 14 days interval thereafter. The cumulative germination data was used in analysis.

In field test, seedbed was prepared by repeated digging and mixing well decomposed farmyard manure at the rate of 10 t/ha. Seeds from each sample were sown continuously in single row at two-centimetre depth with row to row spacing of 15 cm and in between the replication spacing of 50 cm. Each seed sample was sown in four replicates of 1000 seeds each. The seed bed was mulched for the first two weeks. The number of seedlings for each treatment was recorded at eight weeks.

### Seed viability examination

A total of 100 bold seeds comprising 25 seeds/replicate of each seed sample was used for viability test. The tetrazolium solution was prepared by adding 0.1 g of triphenyl tetrazolium chloride (> 95%) into 100 ml of autoclaved distilled water in a beaker. The solution in beaker was wrapped by aluminium foil, stored at 4 °C and used within one week of preparation.

Seeds for viability test were placed in between two layers of paper towel, moistened with 2 ml of sterile water, transferred into a Ziploc bag, and incubated at 20 °C for 18 h. Seeds were cut longitudinally with scalpel blade under a dissecting microscope in a way that reveals the embryo cut in two equal halves. One half of each seed for each replicate was stained with 2 ml of 0.1% tetrazolium solution, covered with aluminium foil and incubated at 40 °C for 4 h. Then seeds were examined under a dissecting microscope for embryo staining. The embryo-coloured deep pink or red were recorded as viable and other colours such as brown, black, and whitish were recorded as non-viable.

A subset of 100 bold seeds from the seeds used for viability testing comprising 25 seeds per replication were sown in 15 cm pots filled with soil and compost mixture (2:1 volume/volume) in greenhouse for germination test. All pots were bottom watered, total number of seedlings was counted every week for eight weeks, and cumulative seed germination data was compared with seed viability tests data.

### Experimental data analysis

A statistical software XLSTAT was used for the data analysis^27^. Data on thousand grain weight, caryopsis number, caryopsis weight, seed germination, and viability tests were subjected to analysis of variance (ANOVA). Where treatment effect was significant, treatment means were separated using Duncan's Multiple Range Test (MRT). A linear regression analysis was performed to observe the relationship between caryopsis number and caryopsis weight, and the relationships between seed germination and seed viability.

## Results

### One thousand grains weight

**Figure 2:**
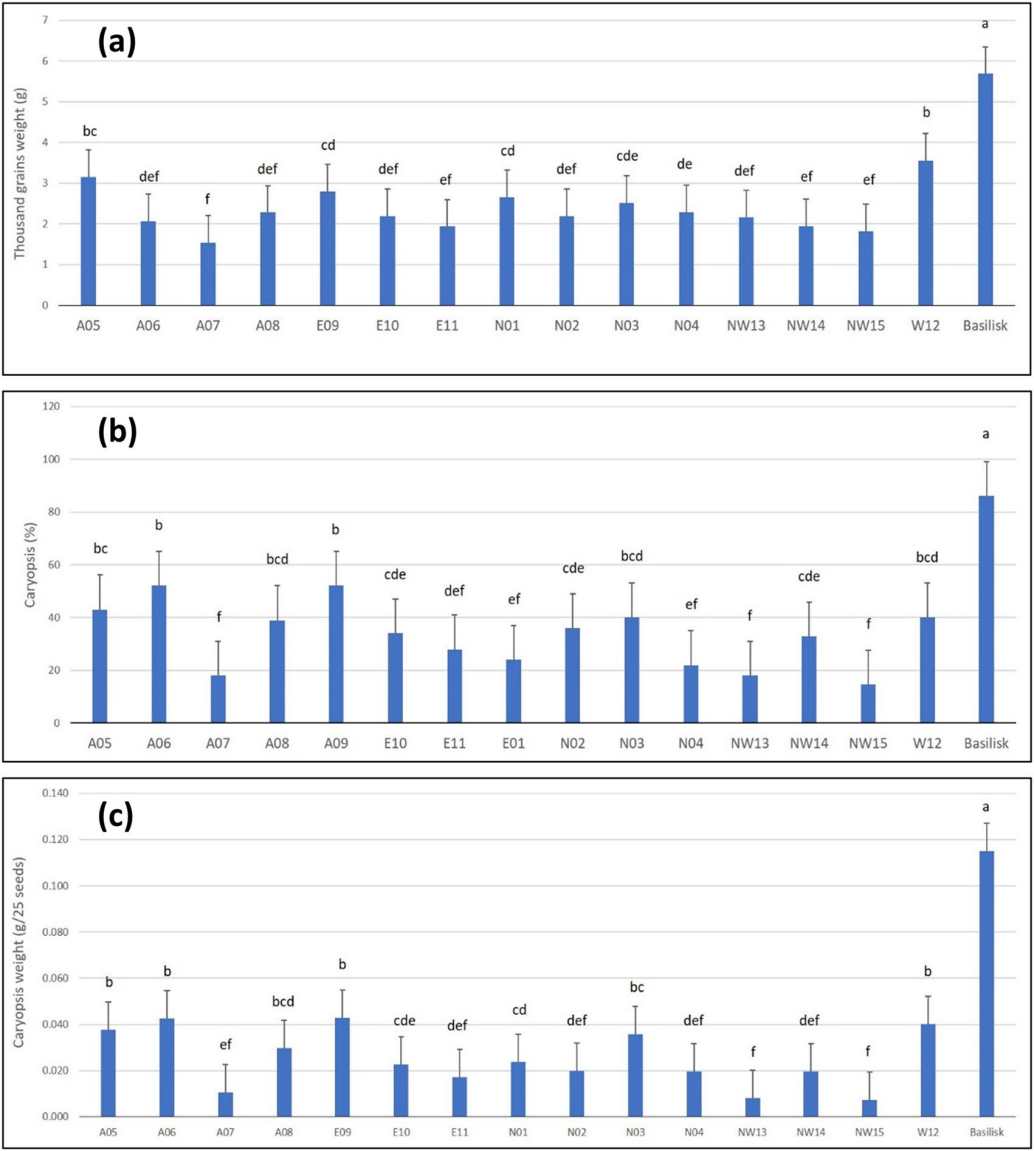
Quality attributes of 15 *Urochloa* seeds samples from Cameroon and seeds of *Urochloa decumbens* cv. Basilisk from Brazil, (**a**) thousand grain weight, (**b**) caryopsis percentage, and (**c**) caryopsis weight. Bars with different letters for each quality attribute are significantly different (*P* < 0.0001). The error bars are standard errors (SE).

The seed samples deferred significantly for TGW (*P* < 0.0001). The TGW ranged from 1.538 g for seed sample A07 to 5.685 g for Basilisk with the mean of 2.549 g (Fig. 2a). Among the *Urochloa* seed samples from Cameroon W12 (3.555 g) and A05 (3.153 g) had the highest TGW.

### Caryopsis percentage

*Urochloa* seed samples were significantly different for the caryopsis formation (*P* < 0.000). Caryopsis formation was highest in Basilisk (86.0%) and lowest in NW15 (14.7%) with the mean caryopsis value of 36.5%. Among the Cameroonian seed samples, the highest caryopsis formation was recorded in A06 and A09, both with caryopsis formation of 52% (Fig. 2b).

### Caryopsis weight

As for TGW and caryopsis formation, seed samples differed significantly for caryopsis weight which ranged between 0.007 g/25 seeds (NW15) to 0.115 g/25 seeds (Basilisk) with mean of 0.031 g/25 seeds (*P* < 0.0001) (Fig. 2c). Simple linear regression analysis of caryopsis percentage and caryopsis weight showed significant and highly positive correlation between these two parameters (r = 0.951; *P* < 0.0001) and caryopsis percentage accounted for 90% of the variability in caryopsis weight.

### Seed germination

In the laboratory, treatment differed significantly for germination which ranged in between zero (N3 and NW15) to 18% (Basilisk) with mean germination of 3.28% (Table 2). Among the seed samples from Cameroon, the highest germination was recorded in N01 (9.75%) and A05 (7.25%).

**Table 2:** Germination of *Urochloa* grass seed samples from different regions of Cameroon at laboratory, greenhouse, and field conditions.

Sample ID	Region	Site	Percent seed germination		
			Lab	Greenhouse	Field
A05	Adamaoua	Wakwa	7.25 bc	14.50 bc	9.24 c
A06	Adamaoua	Banyo	3.25 de	9.25 de	3.48 def
A07	Adamaoua	Banyo	0.50 de	2.25 fg	0.90 g
A08	Adamaoua	Banyo	4.00 cd	8.00 e	4.68 d
E09	East	Bertoua	0.25 de	2.25 fg	3.66 def
E10	East	Bertoua	1.00 de	3.50 fg	1.56 fg
E11	East	Bertoua	2.00 de	3.25 fg	3.00 defg
N01	North	Garoua	9.75 b	18.50 b	15.12 b
N02	North	Louguere	0.50 de	2.00 fg	1.62 fg
N03	North	Louguere	0.00 e	0.75 g	1.56 fg
N04	North	Louguere	1.00 de	2.50 fg	1.08 g
NW13	Northwest	Bambui	0.50 de	5.50 ef	4.26 de
NW14	Northwest	Bambui	1.40 de	5.40 ef	3.74 de
NW15	Northwest	Bambui	0.00 e	2.33 fg	2.08 efg
W12	West	Baganté	3.00 de	12.75 cd	7.68 c
Basilisk	–	–	18.00 a	25.50 a	25.20 a
Mean			3.275	7.390	5.554
Probability			< 0.0001	< 0.0001	< 0.0001

Values with different letter(s) within a column are statistically difference.

As observed in laboratory, seed samples varied significantly for seed germination in greenhouse (Table 2; *P* < 0.0001). The seed germination ranged in between 0.75% (N03) to 25.50% (Basilisk) with mean germination of 7.39%. From 15 seed samples studied, N01 (18.50%) and A05 (14.50%) had the highest germinations rates.

There was significant difference among the seed samples in field germination (Table 2). The germination rate was the highest in Basilisk (25.20%) and was the lowest in A07 (0.90%) with mean germination of 5.55%. The seed samples N01 (15.12%) and A05 (9.24%) had highest germinations rates among the seed samples from Cameroon.

### Seed viability tests

**Figure 3:**
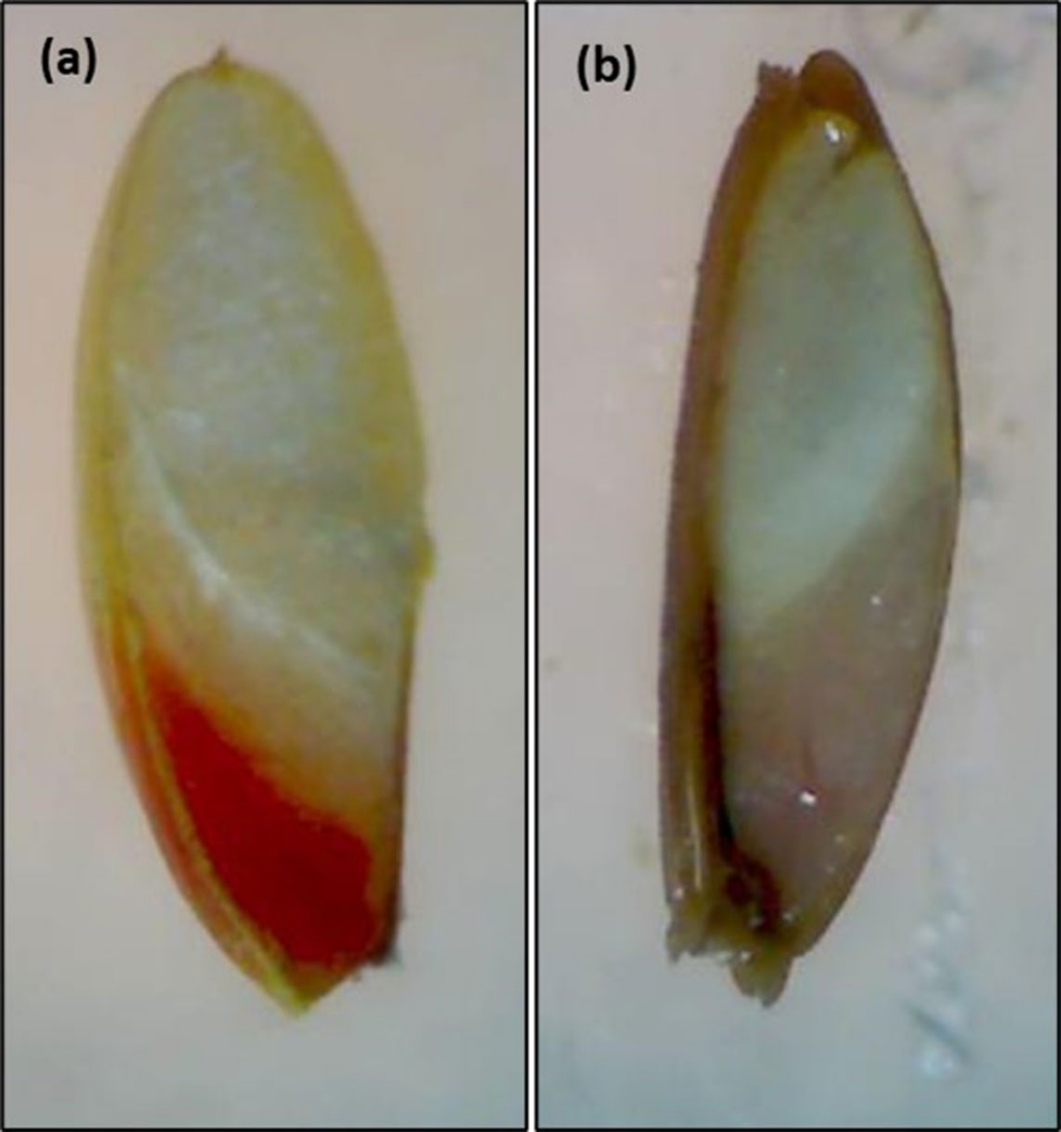
*Urochloa* grass seeds stained with 0.1% tetrazolium solution, (**a**) viable seed showing red embryo, and (**b**) dead seed showing brown embryo.

**Figure 4:**
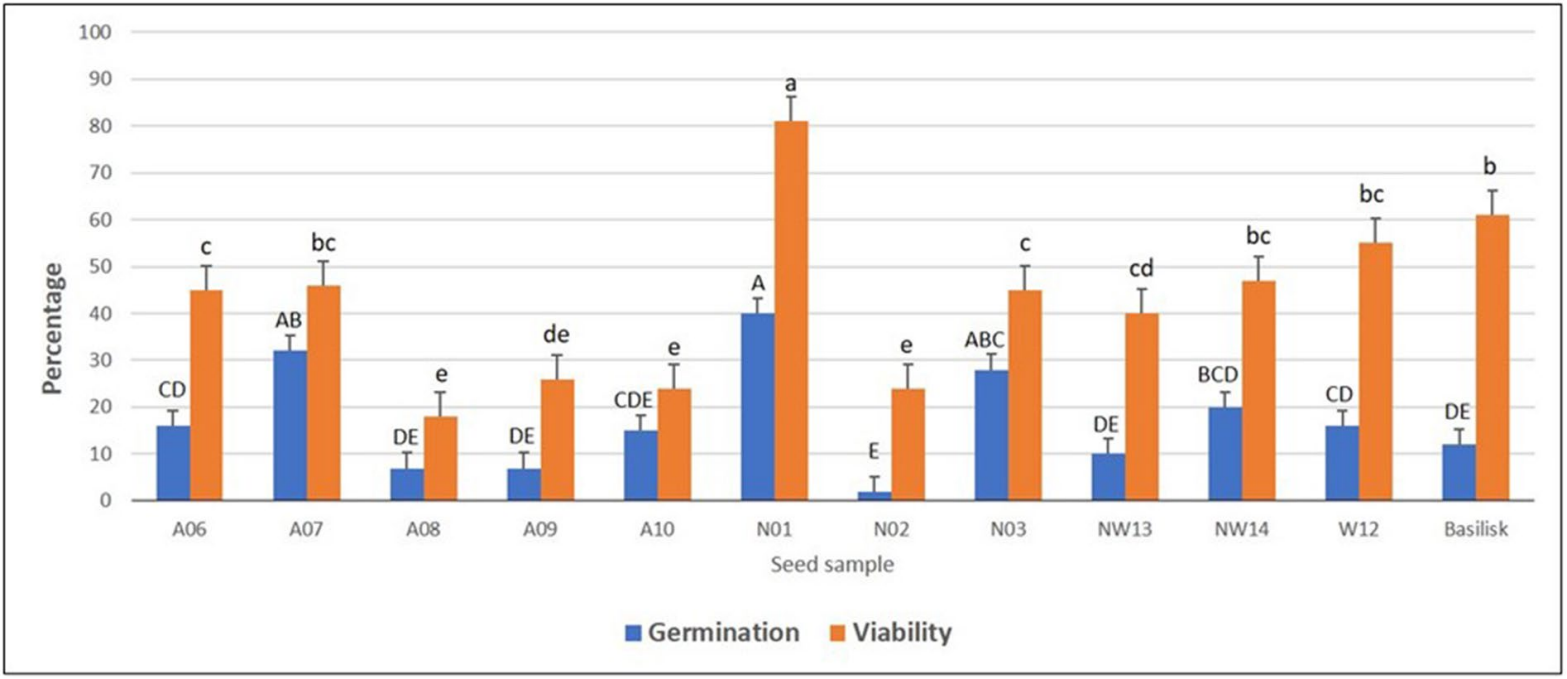
Germination and viability percentage for 12 *Urochloa* spp. seeds samples from Cameroon and seeds of *Urochloa decumbens* cv. Basilisk from Brazil. Bars of same colour with different letters are significantly different (*P* < 0.0001). The error bars are standard errors (SE).

**Figure 5:**
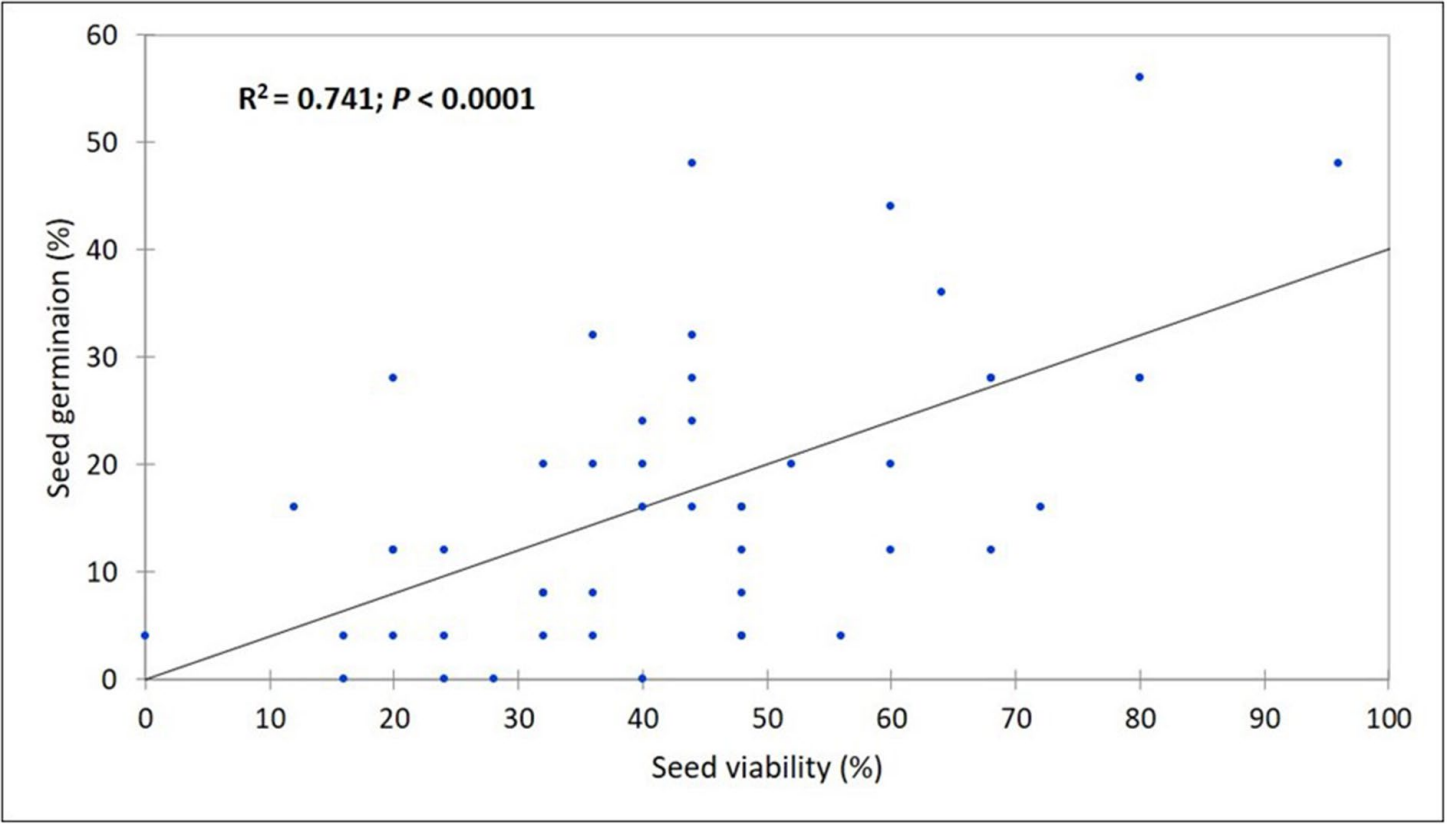
Simple linear regression analysis of seed germination and seeds viability traits showing a significant and positives relationship between these two traits.

The tetrazolium test was effective in differentiating between viable and nonviable *Urochloa* grass seeds (Fig. 3). A significant difference was observed among the 12 seed samples tested for viability (Fig. 4). The seed viability ranged from 18% in E09 to 81% in N01 with mean viability of 42.67%. The viability of improved *U. decumbens* cultivar Basilisk was 61%. The subsets of seed samples tested for viability were also tested for germination. The germination test showed average germination of 17.08% with a range of 2% (N02) to 40% (N01). The differences among the seed samples for germination were significant. A simple regression analysis of seed germination and viability tests data showed positive and significant association between these two parameters (Fig. 5).

## Discussion

In this study 15 *Urochloa* grass seeds samples were collected from Adamaoua, East, North, Northwest, and West regions of Cameroon, and analyzed for quality parameters along with the imported seeds of *Urochloa decumbens* cv. Basilisk from Brazil. As expected, imported Basilisk seeds were the best for almost all seed quality parameters examined. There was clear differences in seed quality attributes among the seed samples originating from different parts of Cameroon suggesting superiority of one site over another for seeds production.

The TGW is one of the important seed quality attributes that has direct relationships with seedling vigor, seedling growth, grain weight and other traits related to kernels and grain yield^28–30^. The variations in TGW within and between the cultivars of a crop are attributed to natural variation, human imposed selections^31^ and seed production environment. The seed samples analyzed in this study were significantly different for TGW. As expected, Basilisk seeds from Brazil had the highest TGW (5.685 g), and the best performing seeds samples from Cameroon i.e., W12 and A05 had TGW of 3.555 g and 3.153 g, receptively. The TGW for Basilisk recorded in the present study was comparable to previous study reporting TGW of 6.846 g for *U. decumbens*^32^. The TGW of 1.659 g, 5.581 g, 8.920 g and 8.600 g have been reported for *U. brizantha*, *U. ruziziensis*, Mulato and Mulato II respectively^32,33^. The difference in TGW for W12 and A05 (which are likely to be *U. ruziziensis*) and TGW reported for *U. ruziziensis* could be explained by the inclusion of empty seeds in our studies while recording TGW^32^. A study in tropical Savanna region of Brazil reports TGW of 2.94 to 4.28 g for *U. humidicola* and 1.78 to 4.77 g for *U. decumbens* cv. Basilisk depending on the harvest and year of harvest^34^. In general, we noticed a low TGW for the most seed samples from Cameroon which could be attributed to inclusion of empty seeds while recording TGW, difference in seed production practices, processing, and storage methods and variations in the seeds production environment.

The caryopsis percentage and caryopsis weight are other important seed quality traits that affect germination, vigour, plant growth as well as the crop performance. We observed marked difference among seed samples analysed in this study for caryopsis percentage and caryopsis weight. Basilisk had the highest caryopsis percent (86%) whereas it ranged in between 17 and 52% for the seed samples from Cameroon suggesting 48–83% of empty seeds that were unable to germinate and produce seedlings. These results were within the range (0–93%) reported for 31 *Urochloa* grass accessions of five *Urochloa* species^35^. The thousand caryopsis weight (TCW) computed for Basilisk (4.400 g) in our study was comparable to *U. decumbens* (4.082 g), and the TCW calculated for two best performing seed samples from Cameroon i.e., E09 and A06 (1.680 g for each) was higher than TCW reported for *U. brizantha* (0.948 g) but was lower than *U. ruziziensis* (4.542 g)^32^.

We used tetrazolium test to determine viability of *Urochloa* seeds, a test commonly used for rapid assessment of seed germination potential. The viability test provides information on number of live seeds and dormant seeds and it is also useful to determine level of damage on seeds during harvesting, processing, and storage. We observed differences among seeds samples for seed viability; three seed samples from Cameroon (A08, NW14 and W12) had similar viability to Basilisk seeds, and N01 had significantly higher seed viability than Basilisk (81% *vs.* 61%). In this study, mean viability was 2.5 folds higher than mean germination (42.67% vs. 17.08%) and as anticipated every seed sample had higher viability than germination (Fig. 4). Similar differences in seed germination percent (5–20%) and corresponding seed viability percent (70–90%) have been reported in some tropical pasture species^36^. A 2.5 folds differences between viability and germination percentages observed in our study could be explained by dormancy imposed by seed covering and embryo dormancy^37,38^. Indeed, all the seed samples from Cameroon were collected in the late 2017 and tested for viability and germination within first 15 months of harvest which may not have been sufficient period for overcoming seed dormancy^39^. The regression analysis revealed 74% variation in seed germination was accounted by seed viability suggesting 26% of variation coming from other factors including seed dormancy.

Seed germination test provides information on ability of the seed to emerge from the soil to produce a plant in the field under normal conditions. The initial seed germination percentage for a quality seed batch should exceed 85% but for plant species that do not reach high levels of germination, a lower germination parentage is acceptable^40^. In this study were performed germination tests in three environments i.e., laboratory, greenhouse, and field; the mean germination rate was the highest in greenhouse test which was followed field test and laboratory test. Except two landraces, the seed germination was consistently high in the greenhouse test. Basilisk seeds had the highest germination (25.5%), and germination for 15 seed samples from Cameroon ranged in between 0.75% to 18.5%. A study in India with four *Urochloa* species reported germination rates from 0.0% to 18.0%^32^. The lowest seed germination rate observed in the laboratory conditions in our study could be attributed to the germination test conditions i.e., constant temperature of 28 °C and continuous dark while other authors have reported the use of alternating day/night temperature and light in assessing seed germination of Brachiaria grass seeds^32,41,42^. We therefore, acknowledge some methodological deficiencies in the lab testing which was solely because of unavailability of incubator with provision of alternate light and temperature cycles. Noteworthy to mention here is that some authors have reported no difference between light and dark conditions for the germination of *U. brizantha* cv. Piata seeds^43^ and no statistical differences between alternating light/dark cycle and continuous dark condition for the germination of *Brachiaria eruciformis* seeds^42^.

Over all, a low germination rates of *Urochloa* seeds observed in our study and other studies could be attributed to various factors including immature embryo at the time of seed dispersal, post-harvest dormancy, storage conditions and length of storage period^33,37,44,45^. Use of about one year old seeds stored in ambient condition might have significantly contributed to a low germination. The practices including seed scarification, seed stratification, exposure of seeds to light, exogenous application of chemical stimulants, invigoration treatments, seed storage in a cool room and use of dry chain technologies including hermetic bags can be helpful to improve seed germination in *Urochloa* grass^37,41,46–49^. Several technologies are available to improve seed germination and other seed quality parameters but their use in many countries in Africa including in Cameroon particularly for tropical forage seed are rare/nonexistence due to limited awareness, low technical know-how, limited access to agricultural inputs and credits, low priority given to forage research and development activities, and subsistence nature of forage and forage seed production. *Urochloa* seeds in Cameroon are harvest during the dry months of November and December enabling farmers to harvest seeds at adequately safe moisture level. These seeds are stored in jute bags on the bamboo ceilings or earthen floors of houses at ambient conditions until June-July, the planting season of *Urochloa* grass). Any unused/surplus seeds are stored further for the next year planting. The quality of these seeds in the ambient storage conditions deteriorate over time because of exposure to high temperature, high humidity and fluctuations in temperature and humidity. The use of technologies like storage of seeds in hermetic super bags and in a cold chain could slow down deterioration of these seeds^41,47^.

The seed production potentials and seed quality attributes including dormancy and germination behaviors of tropical grass species are greatly affected by site characteristics and the prevailing climatic conditions^50^ as they play significant roles in plant growth, flowering, and seed setting. A suitable locality for *Urochloa* seed production should allow unrestricted crop growth with day length that satisfy photoperiod need for flowering and seed setting as well as should restrict continuous vegetative growth to reproductive phase particularly in long day taxa^39^. Such conditions prevail in between about the tenth parallel and Tropic of Cancer in either Hemisphere where most successful *Urochloa* seed production activities have been taking place^35,38,44^. *Urochloa* seed samples from Cameroon analyzed in this study were collected from latitude ranges in between 4.33820°N to 9.93060°N, and seeds samples with the highest germination i.e., N01, A05 and W12 were from Garoua (9.93060°N), Wakwa (7.13600°N) and Bangangte (5.10389°N), respectively. While looking at other seed quality parameters e.g., TGW and caryopsis traits, seeds from higher latitudes were not necessarily superior to seeds from the lower latitudes which could be explained by the difference between the sites in other factors such as soil fertility, amount of rainfall, and altitude^39^.

The five *Urochloa* seeds samples from Cameroon with the highest germination had germination in between 8.0 and 18.5% which was 31.4% to 72.5% of improved cultivar Basilisk from Brazil. In general, the seed germination rates observed in our study was low both for seed samples from Cameroon and Basilisk seeds. This could have been attributed to a low seed setting in many tropical forage species^51^, inherent dormancy in fresh seeds (for seed samples from Cameroon), and loss of germination of Basilisk seeds due to an extended storage period. The observed differences for seeds quality traits among the *Urochloa* seeds samples from Cameroon could be due to the agroclimatic differences among seeds production localities and the variations in practices among farmers in production, processing, and storage of those seeds.

## Conclusions

Farmers in Cameroon mostly from the Adamaoua and North regions have been involved in *Urochloa* seed production for years, but no information was available about seed qualities. This study closes existing knowledge gaps about the qualities of *Urochloa* seeds produced in Cameroon. Our study showed marked differences among seed produced in different localities and regions of Cameroon for major seed quality parameters—thousand gains weight, caryopsis formation, caryopsis weight, seed germination and seed viability. *Urochloa* seeds from Garoua (North), Wakwa (Adamaoua), and Baganté (West) were of superior quality to seeds produced in other localities for most seed quality parameters assessed in the study suggesting possibility of exploring these localities and regions further for *Urochloa* seeds production at commercial scale. Mapping *Urochloa* seed producers in North, Adamaoua and West regions; quality analysis of the seeds from across these three regions; documentation of biophysical characteristics of seed production localities; understanding of farmers’ practices of production, processing, and storage of seeds; and the seed agronomy research are some areas suggested for future research that help to comprehend full potential of Cameroon for the commercial production of *Urochloa* seeds.

## Data Availability

The data sets used and/analysed in this study are available from the corresponding author.

## Permission for collection of seeds

The first author obtained appropriate permission for the collection of *Urochloa* grass seeds used in this study.
